# Articular varus angles of the elbow are not associated with coronoid fracture type

**DOI:** 10.1016/j.jseint.2026.101724

**Published:** 2026-04-30

**Authors:** Huub H. de Klerk, Jort Wiersma, Catherine Hua, Tijn Versteeg, Inger Sierevelt, Ronald Mercer, Abhiram R. Bhashyam, Job N. Doornberg, Neal C. Chen, Michel P.J. van den Bekerom

**Affiliations:** a Department of Orthopedic Surgery, Massachusetts General Hospital, Boston, MA, USA; b Amsterdam Shoulder and Elbow Center of Expertise (ASECE), OLVG, Amsterdam, The Netherlands; c Department of Orthopaedic Surgery, University Medical Centre Groningen (UMCG) and Groningen University, Groningen, The Netherlands; h Harvard Medical School, Boston, MA, USA; d Xpert Clinics, Orthopedic Department, Amsterdam, The Netherlands; e Spaarne Gasthuis Academy, Orthopedic Department, Hoofddorp, The Netherlands; f Department of Orthopaedic Surgery, Amsterdam UMC, Amsterdam, the Netherlands; g Department of Radiology, Massachusetts General Hospital, Boston, MA, USA; i Department of Orthopedic and Trauma Surgery, Flinders University, Adelaide, Australia; j Faculty of Behavioural and Movement Sciences, Vrije Universiteit Amsterdam, Amsterdam, The Netherlands

**Keywords:** Ulnar coronoid process, Bone fracture, Elbow joint, Articular surface angle, Humerus, Computed tomography

## Abstract

**Background:**

The articular surface angle of the elbow (ie, valgus/varus alignment) could affect how traumatic forces are distributed through the ulnohumeral joint, potentially influencing coronoid fracture types. This study hypothesizes that an angle toward varus would be associated with coronoid fractures involving the anteromedial facet (O'Driscoll type 2), rather than fractures limited to the anterolateral tip (O'Driscoll type 1). This study aims to explore the potential association between radiologic elbow varus angles and O'Driscoll coronoid fracture types.

**Materials and methods:**

This retrospective study was conducted at an urban Level 1 trauma center. Adult patients with anterolateral tip or anteromedial coronoid fractures and an elbow computed tomography (CT) scan within four weeks of injury were identified from February 2014 to July 2023, resulting in 142 patients. The mean age was 48 ± 18 years, and 52% of patients were male (74 of 142). Each coronoid fracture was classified independently according to the O'Driscoll classification by 2 trained observers using radiographs, two-dimensional and three-dimensional CT scans with humeral subtractions, and intraoperative findings. Of the patients, 48% (68 of 142) presented with type 1 anterolateral tip fractures, and 52% (74 of 142) with type 2 anteromedial fractures. Interrater reliability of fracture classification was excellent (kappa = 0.90, 95% confidence interval [CI]: 0.83-0.97). Three elbow varus angles were assessed in this study: Trochlear Articular Surface Angle (TASA), Proximal Ulna Articular Surface Angle (PUASA), and Proximal Ulnar Varus Angle (PUVA). Each angle was measured independently by 2 observers using coronal CT scans. Inter-rater reliability was excellent for TASA (intraclass correlation coefficient [ICC] = 0.95, 95% CI: 0.91-0.97), good for PUASA (ICC = 0.89, 95% CI: 0.79-0.91), and moderate for PUVA (ICC = 0.69, 95% CI: 0.56-0.79). Multivariate logistic regression was performed to evaluate the independent association between each varus angle and fracture type, controlling for age, body mass index, gender, hand dominance, and radial head involvement.

**Results:**

The multivariate logistic regression analysis showed no association between the O'Driscoll coronoid fracture types and the TASA (odds ratio [OR]: 0.99; P = .79)’, PUASA (OR: 0.99; *P* = .75), or PUVA (OR: 0.99; *P* = .86).

**Conclusion:**

Occurrence of anteromedial facet fractures is not found to be associated with varus alignment of the elbow in this study. Factors besides the injury mechanism causing translational fractures in the coronoid fracture spectrum should be further explored in future studies to increase our understanding of the etiopathogenesis of the various coronoid fracture types.

The coronoid is an important anterior buttress of the elbow joint that resists varus forces and posteriorly directed forces on the forearm.^6^^,^^8^ However, when these forces become excessive, such as during a fall onto an outstretched hand, the coronoid can fracture. To aid surgical decision-making, coronoid fractures are commonly classified based on fracture morphology using the O'Driscoll classification into type 1: anterolateral tip fracture, type 2: anteromedial facet fracture, and type 3: base fracture.^15^ According to classic teaching, each coronoid fracture morphology is highly associated with a particular injury pattern.^3^

However, a recent study^10^ challenges this common assumption, as coronoid fractures with a concomitant radial head fracture are not consistently coronoid type 1 anterolateral fractures (ie, terrible triad injuries), as per traditional teaching, but about one-third are type 2 anteromedial facet fractures. This suggests that additional factors, beyond the mechanism of injury alone, may influence coronoid fracture pathomorphology. One potential factor could be varus alignment in the elbow, defined by angles such as the Trochlear Articular Surface Angle (TASA), Proximal Ulna Articular Surface Angle (PUASA), and Proximal Ulnar Varus Angle (PUVA) (Fig. 1). Variations in these angles could influence how traumatic forces pass through the ulnohumeral joint, thereby affecting coronoid fracture morphology.^2^^,^^4^^,^^9^^,^^18^ The authors hypothesize that an angle toward varus would be associated with coronoid fractures involving the anteromedial facet (O'Driscoll type 2), rather than fractures limited to the anterolateral tip (O'Driscoll type 1). Understanding the variations in the articular surface angle may advance our understanding of the etiopathogenesis of the various coronoid fracture types (Fig. 2).

**Figure 1:**
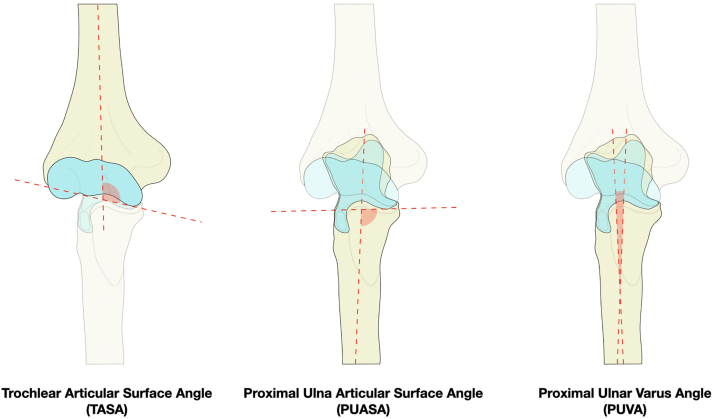
Varus angles of the elbow.

**Figure 2:**
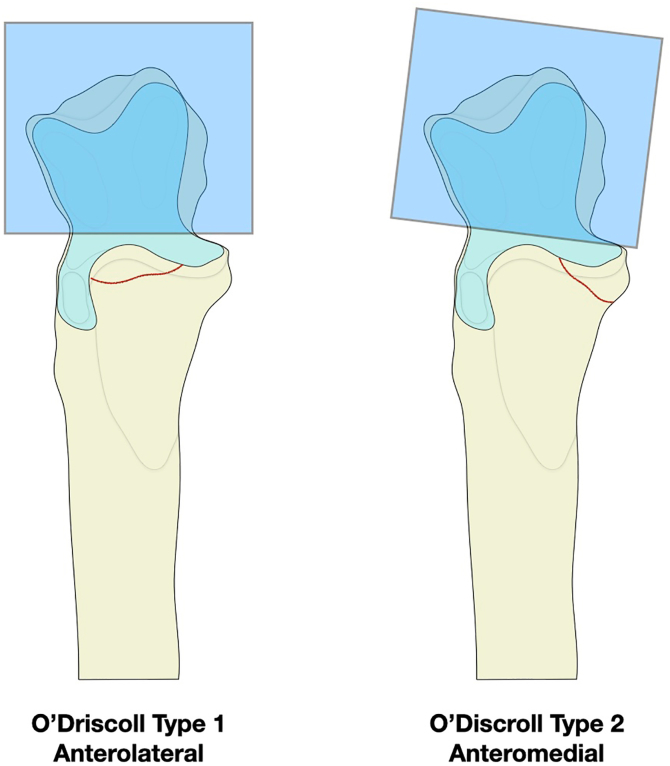
Hypothesis based on angle of traumatic forces.

This study aimed to explore this potential association between radiologic elbow varus angles and the O'Driscoll coronoid fracture types in this retrospective imaging study of a prospective series of coronoid fractures.

## Materials and methods

### Study design and setting

A retrospective imaging analysis was performed based on patients from an urban Level 1 trauma center in North America (Massachusetts General Hospital in Boston, USA) to assess our study's aim to explore the association between the elbow varus angles and fracture types seen in coronoid fractures. The Strengthening the Reporting of Observational Studies in Epidemiology and Sex and Gender Equity in Research guidelines were used.^7^^,^^17^

### Patient selection

The Massachusetts General Brigham database was searched with International Classification of Diseases (ICD) codes and Current Procedural Technology Codes (CPT) for injuries to the elbow and forearm (ICD10: S50-S59) with a computed tomography (CT) scan of the elbow (CPT: 73200-73202 and 73206) between February 2014 and July 2023. During this period, elbow CT scans were typically ordered to evaluate complex fractures, confirm diagnoses when radiographs were inconclusive, assess joint involvement, or plan for surgical procedures. Using the combination of CPT and ICD codes along with the descriptions in the radiology reports, the search identified 6,486 patients.

**Figure 3:**
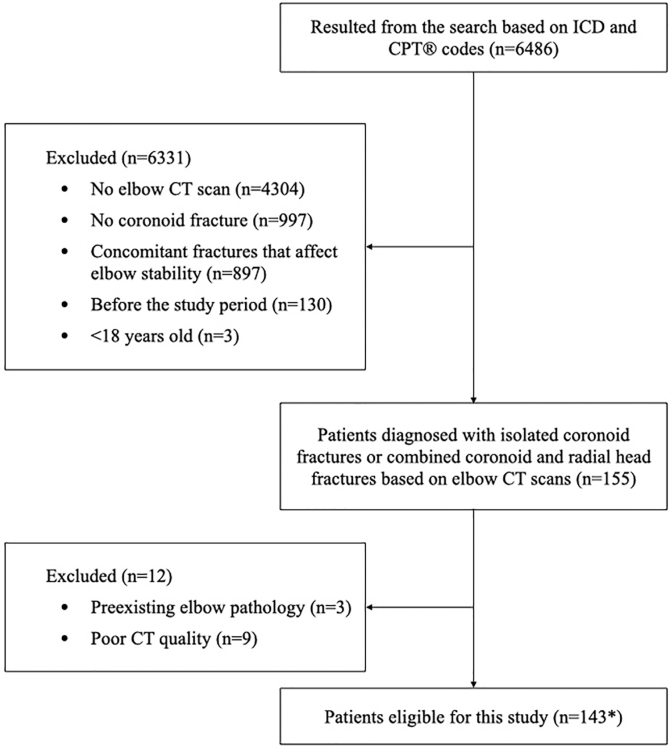
Flowchart of the patient selection process. ∗One patient presented with bilateral coronoid fractures, so this patient was counted twice. *ICD*, International Classification of Diseases; *CPT*, Current Procedural Terminology; *CT*, computed tomography.

Patients were included if they presented with (1) an isolated fracture of the coronoid or combined fracture of the coronoid and radial head; (2) an available elbow CT scan within 4 weeks after the initial radiograph, including visualization of the humeral diaphysis; and (3) age 18 years or older. Based on that, 155 of 6,486 (2.4%) were eligible. Of this eligible cohort, 12 of 155 (7.7%) were subsequently excluded due to pre-existing elbow pathology, prior surgery, or low-quality CT images (including slice thickness over 2 mm, motion artifacts, and incomplete visualization of the osseous structure of the elbow and all its articulations). This resulted in 143 of 155 (92.3%) of eligible patients for analysis. One patient presented with bilateral coronoid fractures, so was counted twice (Fig. 3). Following this initial screening, 6 additional patients with basal coronoid fractures (O'Driscoll type 3) were excluded due to the small size of this group, making it unsuitable for statistical testing and irrelevant to the study hypothesis. The sample size was determined based on the availability of eligible patients who met the specified criteria, and all eligible patients were included. The Digital Imaging and Communications in Medicine (National Electrical Manufacturers Association, Rosslyn, VA, USA) files of selected CT scans were obtained through the Picture Archiving Communications System database of the hospital.

### Descriptive data

One hundred forty-three fractures in 142 patients were included and classified. Coronoid fractures were classified using the O'Driscoll classification, with 48% (68 of 142) of the patients presenting with type 1 anterolateral fractures and 52% (74 of 142) with type 2 anteromedial fractures. Among the type 1 fractures, 9% (6 of 68) were subcategorized as subtype 1 (≤2 mm) and 91% (62 of 68) as subtype 2 (>2 mm). For type 2 fractures, 4% (3 of 74) were classified as subtype 1, 88% (65 of 74) as subtype 2, and 8% (6 of 74) as subtype 3. No difference in patient demographics was seen between the O'Driscoll type 1 and 2 fractures besides the involvement of the radial head (77% vs. 49%; *P* = < .001) (Table I). This difference in concomitant radial head involvement likely reflects the predominance of the terrible triad injury pattern in type 1 anterolateral fractures, whereas type 2 anteromedial fractures also occur in posteromedial varus rotational instability patterns in which radial head fractures are less common.^10^^,^^11^ None of the patients identified as nonbinary, gender diverse, or preferred not to disclose their gender.

**Table I:** Patient demographics and fracture characteristics.

Characteristic	Total (n = 142)	Type 1 (n = 68)	Type 2 (n = 74)	*P* Value
Age in yr, mean (SD)	48.2 (17.7)	46.5 (18.3)	49.8 (17.0)	.27
Female, n (%)	68 (48)	33 (49)	35 (47)	.88
BMI in kg/m^2^, mean (SD)	28.5 (6.5)	27.8 (6.6)	29.0 (6.5)	.30
Right elbow, n (%)	69 (49)	33 (49)	36 (49)	.99
Dominant arm, n (%)∗	50 (45)	23 (44)	27 (47)	.81
Concomitant radial head involvement, n (%)	88 (62)	52 (77)	36 (49)	<.001

*n*, number of patients; *SD*, standard deviation; *BMI*, body mass index.

∗ missing n = 32.

### Radiologic assessment

To improve the accuracy of radiologic fracture assessments, precautions were taken before the classification process began. The researchers (HK and JW), who were graduate students with at least three years of medical school and prior orthopedic research experience on the elbow, received training from a fellowship-trained upper extremity orthopedic surgeon (MB) to ensure they could accurately assess the fractures. This training included a review of key characteristics for differentiating coronoid fracture types and the classification of multiple cases that the surgeon had previously used in lectures. The surgeon then reviewed the classifications made by the researchers to confirm their accuracy and understanding. After the surgeon approved their work and was confident in their ability to apply the classification system correctly, the researchers began data collection.

The 2 trained researchers (HK and JW) independently classified the fracture types for each included patient based on radiographs, two-dimensional CT scans, three-dimensional (3D) CT scans, and intraoperative findings under the supervision of a fellowship-trained upper extremity orthopedic surgeon (MB). Coronoid fractures were categorized according to fracture morphology using the O'Driscoll classification into type 1: tip fracture or type 2: anteromedial facet fracture.^15^ The O'Driscoll types were then further subcategorized.^15^ Type 1 was subcategorized as ≤2 mm (subtype 1) and >2 mm coronoid tip fractures (subtype 2). Type 2 was subcategorized as fractures involving the anteromedial rim (subtype 1), anteromedial rim plus tip (subtype 2), and anteromedial rim plus sublime tubercle with or without the involvement of the tip (subtype 3). Coronoid fractures were evaluated using axial, coronal, and sagittal CT slices, 3D CT reconstructions with humeral subtractions, along with intraoperative findings. The inter-rater reliability of the radiologic O'Driscoll classification was assessed using Cohen kappa, which yielded an unweighted kappa value of 0.90 (95% confidence interval [CI]: 0.83-0.97), with an absolute agreement of 95% (136 of 143), indicating a strong level of agreement between the raters.^13^^,^^14^ When there was no consensus among the researchers regarding the fracture type, discussions were held with a fellowship-trained upper extremity orthopedic surgeon (MB) to reach an agreement.

**Figure 4:**
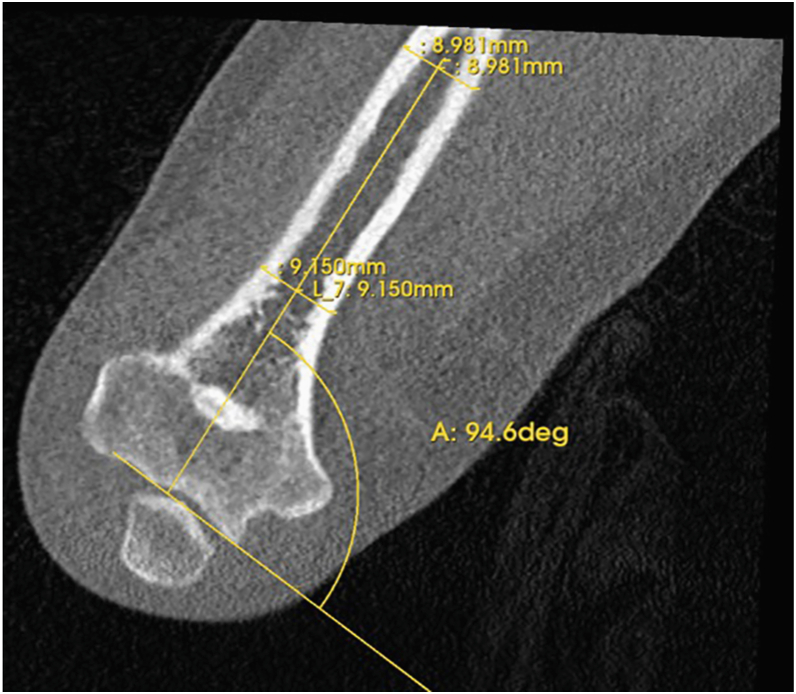
TASA measurements. *TASA*, Trochlear Articular Surface Angle.

In this study, 3 varus angles of the elbow were defined: TASA, PUASA, and PUVA (Fig. 1). The TASA was defined as the angle between the humerus shaft's longitudinal axis and a transverse line along the most distal aspects of the trochlea (Fig. 4). The PUASA was defined as the angle between the proximal ulna shaft's longitudinal axis and a transverse line along the most proximal articular surface of the olecranon. The PUVA represents the diaphyseal varus bend of the proximal ulna and was defined as the angle between the shaft axis and proximal-segment axis. The specific instructions for the researchers per angle are presented in Supplementary Data 1. All angles were assessed using a coronal CT scan of the elbow. To ensure reliability, 2 of 3 researchers (JW, CH, TV) independently measured the angles for each included patient. Due to hardware incompatibility, one researcher (CH) used Horos and the other (JW) used 3D Slicer (version 5.6.2; Brigham and Women's Hospital, https://www.slicer.org) for the TASA measurements. For the PUASA and PUVA measurements, both researchers (JW and TV) used 3D Slicer. PUASA and PUVA could not be measured in 20 elbows because the CT did not include sufficient ulnar shaft to construct a longitudinal axis. The inter-rater reliability for measuring the angles was evaluated using the two-way intraclass correlation coefficient (ICC).^12^ For the TASA, the interrater reliability was excellent (ICC = 0.95, 95% CI: 0.91-0.97). The mean interrater difference was 1.1 ± 0.9° (range, 0.03-4.3), and the smallest detectable difference was 2.88°. For the PUASA, the inter-rater reliability was good (ICC = 0.89, 95% CI: 0.79-0.91). The mean interrater difference was 2.2 ± 1.8° (range, 0-8.8), and the smallest detectable difference was 5.51°. For the PUVA, the inter-rater reliability was moderate (ICC = 0.69, 95% CI: 0.56-0.79). The mean inter-rater difference was 1.6 ± 1.1° (range, 0-5.5), and the smallest detectable difference was 3.74°. Bland-Altman analyses were used to assess proportional bias. Visual inspection of the plots (Supplementary Data 2) showed no systematic trend in differences across the angle range, indicating stable agreement between raters over all magnitudes.

### Statistical analysis

As the data were normally distributed, continuous data were reported as mean and standard deviation, and categorical data as numbers with percentages (Supplementary Data 3). A comparison of patient demographics and fracture characteristics was performed using Student *t*-tests and chi^2^-tests. A univariate as well as multivariate logistic regression analysis was employed to assess the association between elbow varus angles and the O'Driscoll coronoid fracture types while controlling for potential confounding variables such as age, body mass index (BMI), sex, hand dominance, and radial head involvement. In our dataset comprising 142 cases, 73% (103 of 142) exhibited complete data across all measured covariates, while missing data were observed in hand dominance (23%) and BMI (11%). For the imputation of these missing data, multiple imputation procedures were applied under the assumption of missing at random using all models and one auxiliary variable (side).^16^ After creating 5 datasets, the calculated odds ratios (ORs) were pooled by using Rubin's rule. A complete case analysis was performed as a sensitivity analysis.

To investigate possible differences between sexes and gender identities, the interaction between gender and angle measurements was assessed. As no patients identified as nonbinary, gender diverse, or declined to disclose their gender, these categories were excluded from our analysis. All patients reported that their gender matched their biological sex, so a unified analysis was conducted. In case of interaction between sex and angle, analysis would be stratified for sex. The significance of differences between values, including age and BMI, was calculated using the independent samples t-test. The significance of differences between frequencies, including sex, injured side, hand dominance, and radial head involvement, was calculated using the chi-square test. Because angle distributions were approximately normal, pairwise associations were assessed between TASA, PUASA, and PUVA using Pearson correlation with pairwise deletion, reporting 95% CIs.

Statistical analyses were performed using Statistical Package for the Social Sciences (IBM Corp. Released 2023. IBM SPSS Statistics for Windows, Version 29.0.2.0 Armonk, NY: IBM Corp), and statistical significance was set at a *P* value of less than .05.

## Results

### Trochlear articular surface angle

The mean TASA for the entire cohort was 102.5 ± 4.1° (range, 87.3-112.0). Patients with type 1 fractures, limited to the anterolateral aspect, had a mean angle of 102.6 ± 3.5°. Those with type 2 fractures (involving the anteromedial aspect) had a mean articular surface angle of 102.4 ± 4.5°.

Both crude and adjusted ORs showed no significant association between the radiologic TASA and O'Driscoll coronoid fracture types, with values of 0.96 (95% CI: 0.86-1.07; *P* = .43) and 0.99 (95% CI: 0.90-1.08; *P* = .79), respectively. Sensitivity analysis revealed a comparable OR of 0.98 (95% CI: 0.85-1.12; *P* = .72). No interaction was found between sex and TASA (*P* = .98), so further stratification by sex was not conducted.

### Proximal ulna articular surface angle

The mean PUASA for the entire cohort was 97.8 ± 5.0° (range, 87.0-110.5). Patients with type 1 fractures had a mean angle of 97.9 ± 5.4°. Those with type 2 fractures had a mean articular surface angle of 97.8 ± 4.7°.

Both crude and adjusted ORs showed no significant association between the radiologic PUASA and O'Driscoll coronoid fracture types, with values of 0.99 (95% CI: 0.92-1.06; *P* = .75) and 0.98 (95% CI: 0.90-1.07; *P* = .66), respectively. Sensitivity analysis revealed a comparable OR of 0.99 (95% CI: 0.92-1.07; *P* = .87). No interaction was found between sex and PUASA (*P* = .49).

### Proximal ulnar varus angle

The mean PUVA for the entire cohort was 11.4 ± 2.3° (range, 6.6-17.5). Patients with type 1 fractures had a mean angle of 11.4 ± 2.6°. Those with type 2 fractures had a mean articular surface angle of 11.5 ± 2.1°.

Both crude and adjusted ORs showed no significant association between the radiologic PUVA and O'Driscoll coronoid fracture types, with values of 0.99 (95% CI: 0.84-1.16; *P* = .86) and 0.99 (95% CI: 0.80-1.21; *P* = .88), respectively. Sensitivity analysis revealed a comparable OR of 0.98 (95% CI: 0.83-1.16; *P* = .84). No interaction was found between sex and PUVA (*P* = .63).

### Intercorrelations of elbow varus angles

PUVA showed no correlation with either TASA (r = −0.10; 95% CI: −0.29 to 0.10; *P* = .313) or PUASA (r = −0.01; 95% CI: −0.21 to 0.18; *P* = .01). TASA and PUASA were weakly, negatively correlated (r = −0.31; 95% CI: −0.47 to −0.12; *P* = .002). Overall, the angles do not appear to scale together as a single global alignment metric, but behave as independent anatomical features.

## Discussion

The association between coronoid fracture types and injury patterns is less strong than traditionally assumed.^10^ This suggests that, beyond the injury mechanism, additional factors may shape the fracture morphology. This study explored patient-specific varus alignment as a potential contributor, as it may affect how traumatic forces are distributed through the ulnohumeral joint. The authors hypothesized that an angle toward varus would be associated with coronoid fractures involving the anteromedial facet (O'Driscoll type 2), rather than fractures limited to the anterolateral tip (O'Driscoll type 1). Despite careful, rotation-controlled CT methods, subtle varus alignment differences alone did not explain the spectrum of coronoid fracture pathoanatomy observed in complex elbow injuries. Intercorrelation findings suggest that the 3 angles capture distinct anatomical features rather than a single global varus alignment construct. These results do not support this hypothesis and suggest that either key determinants are being overlooked or that morphology reflects a combination of factors such as patient-specific osseous anatomy, soft-tissue integrity, and moreover: dynamic conditions at the moment of impact. Investigating these factors could advance our understanding of etiopathogenesis across the spectrum of such fractures.

### Radiologic elbow angles and coronoid fracture type

Our study found no association between the radiologic varus angles and O'Driscoll coronoid fracture types. Although this finding does not result in altered clinical decision-making, it can add to the etiopathogenetic understanding of these fractures by suggesting that subtle variations in ulnar and trochlear morphology do not account for the spectrum of coronoid fracture patterns. This insight encourages clinicians and researchers to look beyond geometric differences of the distal humerus and proximal ulna and to consider other biomechanical or patient-related factors that better explain the different fracture types seen in the same injury pattern.

Arrigoni et al^1^ aimed to analyze humeral trochlear morphology in patients with simple elbow dislocations (n = 25) or complex elbow dislocations with isolated coronoid fractures (n = 37), excluding those with sublime tubercle involvement and transolecranon fracture-dislocations. Although the authors measured different angles (anterior, distal, and posterior trochlear angles) and demonstrated reliable reproducibility (ICC = 0.71-0.88), their findings also showed no association between trochlear morphology and coronoid fracture type in the subgroup with complex elbow dislocations with isolated coronoid fractures. Although Arrigoni et al also investigated the relationship between humeral trochlear morphology and coronoid fracture types, they included 37 patients with complex elbow dislocations, resulting in only 18 O'Driscoll type 2 fractures and even fewer type 1 fractures. Consequently, their statistical power to detect meaningful associations was limited. In addition, our study encompassed all complex elbow patterns involving the anteromedial and/or anterolateral facet of the coronoid, such as posteromedial varus rotational instability injuries and terrible triad injuries, yielding a larger cohort. This expanded range of fracture types and higher sample size allowed us to statistically control for additional confounders (eg, age, BMI, sex, and radial head involvement) to isolate the potential impact of the TASA. As a result, our findings provide a more reliable assessment of the influence of anatomical variations on coronoid fracture types.

Hancerli et al^5^ assessed whether anatomical variations of the proximal ulna influence the occurrence of simple elbow dislocations or complex elbow dislocations. The Proximal Ulna Dorsal Angulation, PUVA, and Olecranodiaphyseal Angle (ODA) on radiographs or 3D-CT were used to quantify anatomical variations of the proximal ulna. Based on 347 participants, the authors found differences in the ODA across healthy elbows, simple elbow dislocations, and complex dislocations. They found that ODA was lower in complex dislocations than in healthy and simple groups. The authors also state that there was no difference in Proximal Ulna Dorsal Angulation, PUVA, and ODA between Regan-Morrey coronoid fracture types, although the sample size of the coronoid fractures was limited (n = 47). Our study extends this work in 2 ways. First, this study focused on coronoid fracture types within complex patterns (posteromedial varus rotational instability and terrible triad), directly testing an etiopathogenetic hypothesis. Second, because PUVA from radiographs or 3D reconstructions is rotation-sensitive, this study used a rotation-controlled coronal CT slice to standardize viewing before measuring TASA, PUASA, and PUVA. Rotation control is important for causal inference because even small rotation errors can overestimate or underestimate the angle, which can mask a true association between factors. Despite these methodological advantages, this study found no association between any varus-angle measure and O'Driscoll fracture type.

### Limitations

There are multiple considerations when interpreting the results of this study. First, only 155 of 6,486 (2.4%) of screened patients met eligibility criteria, reflecting an intentionally broad search designed to capture all potential elbow or forearm injuries with CT in a rare condition and to reduce labeling inconsistencies over 9 years. Of those eligible, an additional 12 of 155 (7.8%) were excluded for pre-existing pathology, prior surgery, or poor image quality, yielding a small but comprehensive and unbiased final cohort. Secondly, transolecranon fracture-dislocations were excluded from our analysis to focus on O'Driscoll coronoid fracture types 1 and 2. Because transolecranon fracture-dislocations are highly associated with O'Driscoll type 3 fractures,^11^ including them would not have contributed to the aim of this study. However, this decision limits our ability to assess all coronoid fracture morphologies comprehensively. Thirdly, TASA and PUASA were defined using the most distal trochlear articular surface and the proximal ulna's articular surface near the coronoid base, assuming contact during elbow extension typical of a fall-onto-an-outstretched-hand (FOOSH) mechanism. Because some of the coronoid fractures might arise from other mechanisms or joint positions, the true load-bearing articular surface may differ, and our angle definitions may not fully reflect those scenarios. Lastly, due to hardware incompatibility, the TASA measurements were performed on 2 different software platforms (Horos by one researcher and 3D Slicer by another researcher), each with its own interface and measurement tools. However, the excellent inter-rater agreement (ICC = 0.95, 95% CI: 0.91-0.97) and Bland-Altman analysis suggest that these platform differences did not affect the reliability or overall outcome of the measurements.

## Conclusion

Our study found no association between the elbow varus angles and O'Driscoll coronoid fracture types 1 and 2. Although this study hypothesized that an angle toward varus would be associated with anteromedial facet fractures (O'Driscoll type 2), our findings indicate that an angle toward valgus or varus alone does not account for the spectrum of coronoid fracture morphologies seen in complex elbow injuries. Other biomechanical or patient-related factors should be further explored in future studies to explain the different coronoid fracture types seen in the same injury pattern.

## Disclaimers:

Funding: This work was in part supported by the Jesse B. Jupiter Research Fund of the Wyss Medical Foundation. NC certifies receipt of personal payments or benefits, during the study period, in an amount of less than USD 10,000 from Biedermann Motech. Additionally, HK reports receipt of support by De Stichting Prof. Michaël-van Vloten Fonds (the Hague, the Netherlands), Van Leersum Grant / KNAW Medical Sciences Fund from the Royal Netherlands Academy of Arts & Sciences (Amsterdam, the Netherlands), Marti-Keuning Eckhardt Foundation (Amsterdam, the Netherlands), Vreedefonds (Amsterdam, the Netherlands), the Stichting Anna Fonds | NOREF (Mijdrecht, the Netherlands), the Stichting het Scholten-Cordes Fonds (the Hague, the Netherlands), Fundatie van Renswoude (‘s-Gravenhage, the Netherlands), and the USC Scholarship Foundation (Utrecht, the Netherlands).

Conflicts of interest: The authors, their immediate family, and any research foundation with which they are affiliated have not received any financial payments or other benefits from any commercial entity related to the subject of this article.
