# Supplementary material for: Articular varus angles of the elbow are not associated with coronoid fracture type
Source: JSES Int. 2026 Apr 30;10(4):101724. doi: 10.1016/j.jseint.2026.101724 (PMC13264352; doi:10.1016/j.jseint.2026.101724)
Supplement: Supplementary Data 1 [file mmc1.docx]

**Supplementary Data 1 – Angle Measurements Tutorial**

Trochlear Articular Surface Angle (TASA)

The coronal view of a patient's CT scan was selected to measure both the transverse line of the trochlea and the longitudinal axis of the humerus. In this view, the most distal point of the medial trochlear ridge was identified. This point became the center of rotation around which the plane was rotated along the axial axis until the most distal point of the lateral trochlear ridge was identified. A line was drawn between these two distal points of the trochlea and named the transverse line. Once drawn, the plane was rotated around the sagittal axis until it went through the center of the humeral shaft. The plane was considered central when the medial and lateral cortex of the humerus ran parallel to each other. All subsequent measurements were made in this plane. The most distal part of the humeral diaphysis was determined. Subsequently, a line was drawn perpendicularly through the medial and lateral cortex of the humerus. This line was divided in half, identifying the exact center of the distal diaphysis. The same steps were taken for the most proximal part of the humeral diaphysis. Once these two central points were determined, a line was drawn through them. This was considered the longitudinal axis of the humerus. The TASA was determined by measuring the angle between the transverse line and the longitudinal axis.

Proximal Ulna Articular Surface Angle (PUASA) and Proximal Ulnar Varus Angle (PUVA)

The measurement of both the PUASA and PUVA angles started in the axial view of the ulna. The slice just distal of the termination of the coronoid was selected. Here a line was drawn through the most volar and dorsal parts of the ulnar cortex. The view was rotated along the axial axis to match this line thereby locking in the axial rotation for all subsequent measurements. Following this, the sagittal view was selected. This view was rotated along the sagittal axis until it was parallel with the dorsal cortex of the ulna. This ensured that all subsequent measurements were taken in a plane that was parallel to the ulnar cortexes. Finally, the coronal view was selected. The slice that was most superior through the coronoid, while being below the coronoid fracture line, was selected. In this plane, a transverse line was drawn between the most lateral point of the coronoids articular surface and the most distal point of the coronoids articular surface. These points articulate with the humerus’ two most distal points in full extension of the elbow. Therefore, a line drawn between these coronoid points will directly match the distal humerus line drawn for the TASA measurements. Once the transverse coronoid line was established, we moved dorsally through the CT slices until a slice with parallel medial and lateral ulnar cortexes was detected. Here two transverse lines were drawn through the ulna. One line was drawn through the most proximal part of the ulna, and the other was drawn just proximal of the anatomical ulnar varus angulation. Both lines were drawn perpendicular to the cortex at the point where the medial and lateral ulnar cortexes were parallel. Similar to the TASA measurements, the midpoints of both lines were determined and connected to form the proximal ulnar line. Following this, the distal ulnar line was established in the same manner, using two transverse reference lines. One line was drawn just distal of the anatomical ulnar varus angulation, and the other was drawn through the most distal aspect of the ulna. The midpoints of both lines were connected to form the distal ulnar line. The PUASA was determined by measuring the angle between the transverse coronoid line and the proximal ulnar line. Finally, the PUVA was determined by measuring the angle between the proximal and distal ulnar lines.
