# Supplementary material for: Articular varus angles of the elbow are not associated with coronoid fracture type
Source: JSES Int. 2026 Apr 30;10(4):101724. doi: 10.1016/j.jseint.2026.101724 (PMC13264352; doi:10.1016/j.jseint.2026.101724)

**Supplementary Data 3 – Distribution of angle measurements**

Trochlear Articular Surface Angle (TASA); the red line represents the mean.


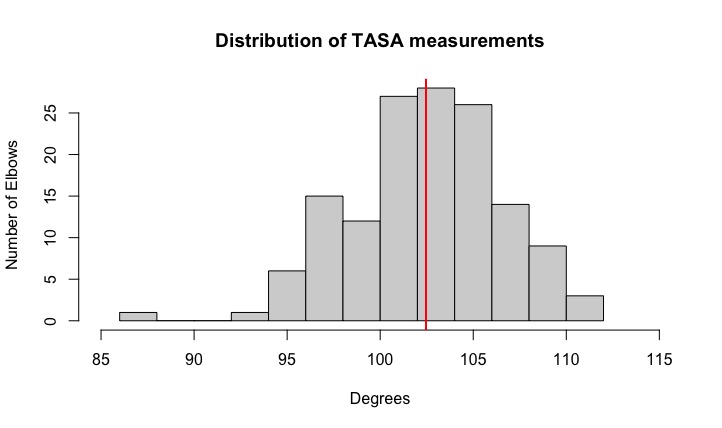


Proximal Ulna Articular Surface Angle (PUASA); the red line represents the mean.


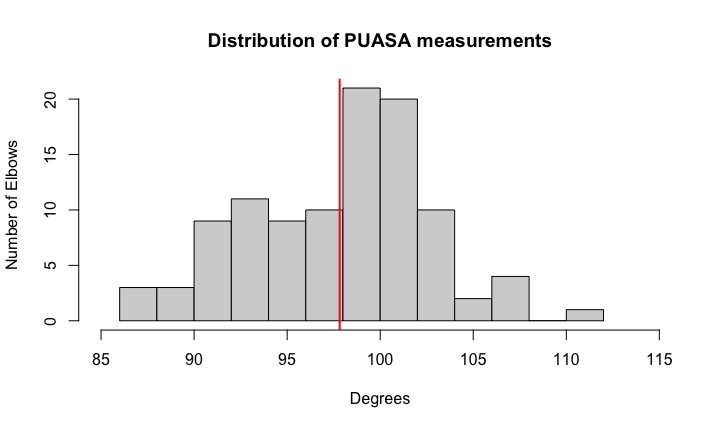


Proximal Ulnar Varus Angle (PUVA); the red line represents the mean.


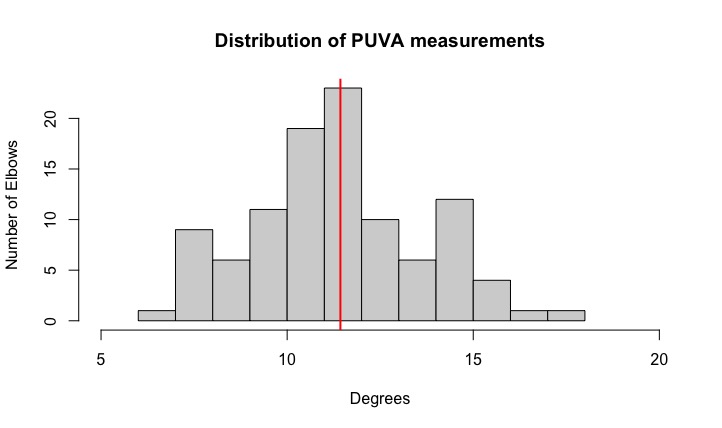

Supplement: Supplementary Data 3 [file mmc3.docx]
